# *Trypanosoma rangeli* Genetic, Mammalian Hosts, and Geographical Diversity from Five Brazilian Biomes

**DOI:** 10.3390/pathogens10060736

**Published:** 2021-06-11

**Authors:** Maria Augusta Dario, Márcio Galvão Pavan, Marina Silva Rodrigues, Cristiane Varella Lisboa, Danilo Kluyber, Arnaud L. J. Desbiez, Heitor Miraglia Herrera, André Luiz Rodrigues Roque, Luciana Lima, Marta M. G. Teixeira, Ana Maria Jansen

**Affiliations:** 1 Laboratório de Biologia de Tripanosomatídeos, Instituto Oswaldo Cruz, Fiocruz, Rio de Janeiro 21040-360, Brazil; maria.dario@ioc.fiocruz.br (M.A.D.); marina.rodrigues@ioc.fiocruz.br (M.S.R.); crisvarella@ioc.fiocruz.br (C.V.L.); roque@ioc.fiocruz.br (A.L.R.R.); 2 Laboratório de Mosquitos Transmissores de Hematozoários, Instituto Oswaldo Cruz, Fiocruz, Rio de Janeiro 21040-36, Brazil; mgpavan@ioc.fiocruz.br; 3 Associate Researcher, Naples Zoo at Caribbeans Gardens, Naples, FL 34102, USA; dkluyber@live.com; 4 Instituto de Conservação de Animais Silvestres (ICAS), Campo Grande 79037-100, Brazil; adesbiez@hotmail.com; 5 Pós-Graduação em Ciência Ambientais e Sustentabilidade Agropecuária, Universidade Católica Dom Bosco, Campo Grande 79117-900, Brazil; herrera@ucdb.br; 6 Pós-Graduação em Ecologia e Conservação, Universidade Federal de Mato Grosso do Sul, Campo Grande 79117-900, Brazil; 7 Departamento de Parasitologia, Instituto de Ciências Biomédicas, Universidade de São Paulo, São Paulo 05508-000, Brazil; lulima@usp.br (L.L.); mmgteix@icb.usp.br (M.M.G.T.)

**Keywords:** *Trypanosoma rangeli*, mammals, parasite ecology, lineages, Brazilian biomes

## Abstract

*Trypanosoma rangeli* is a generalist hemoflagellate that infects mammals and is transmitted by triatomines around Latin America. Due to its high genetic diversity, it can be classified into two to five lineages. In Brazil, its distribution outside the Amazon region is virtually unknown, and knowledge on the ecology of its lineages and on host species diversity requires further investigation. Here, we analyzed 57 *T. rangeli* samples obtained from hemocultures and blood clots of 1392 mammals captured in different Brazilian biomes. The samples were subjected to small subunit (SSU) rDNA amplification and sequencing to confirm *T. rangeli* infection. Phylogenetic inferences and haplotype networks were reconstructed to classify *T. rangeli* lineages and to infer the genetic diversity of the samples. The results obtained in our study highlighted both the mammalian host range and distribution of *T. rangeli* in Brazil: infection was observed in five new species (*Procyon cancrivorous*, *Priodontes maximum*, *Alouatta belzebul*, *Sapajus libidinosus*, and *Trinomys dimidiatus*), and transmission was observed in the Caatinga biome. The coati (*Nasua nasua*) and capuchin monkey (*S. libidinosus*) are the key hosts of *T. rangeli*. We identified all four *T. rangeli* lineages previously reported in Brazil (A, B, D, and E) and possibly two new genotypes.

## 1. Introduction

*Trypanosoma rangeli* (Kinetoplastea: Tripanosomatidae) was first described by Tejera in 1920 [1], infecting the triatomine species *Rhodnius prolixus* (Hemiptera: Reduviidae). This protozoan infects different mammal species, including humans in Latin America and the Caribbean [2,3,4,5,6,7]. Infection is usually considered non-pathogenic to mammals [2,8], but *T. rangeli* induces low-level and long-lasting parasitemia that can last for years [9,10]. Unlike mammals, the parasite is pathogenic to triatomine species of the genus *Rhodnius* [8,11,12]. *Trypanosoma rangeli* and *T. cruzi* usually share mammalian hosts, and their distributions in nature often overlap in simple or mixed infections in mammals and triatomines [4,13,14,15,16]. However, there are marked differences in the transmission routes, life cycles, and host-parasite interactions of these two species. To date, *T. rangeli* metacyclic forms have been found in the salivary glands of only *Rhodnius* species, and parasite transmission by the inoculative route is associated with this triatomine genus [8,17,18,19].

*Trypanosoma rangeli* genetic diversity has been demonstrated by nuclear and mitochondrial markers [4,5,20,21,22,23]. Variability in the kDNA minicircles, as demonstrated by the presence or absence of the KP1 minicircle, separated *T. rangeli* into two groups, called KP1 (+) and KP1 (–) [7,23,24,25,26]. The use of spliced leader rRNA, ITS rDNA, SSU rDNA, and other nuclear sequences showed that *T. rangeli* is composed of two main lineages: one containing lineages A, C, D, and E and the other formed by the phylogenetically basal B lineage [6,27,28,29,30,31,32]. In Brazil, *T. rangeli* is mainly described in mammals and triatomines from the Amazon region; moreover, there are also sporadic reports of its occurrence in the Pantanal, Atlantic Forest, Cerrado, and Caatinga biomes, where it infects different mammal species of the orders Chiroptera, Carnivora, Didelphimorphia, Rodentia, Pilosa, Primates, and Cingulata [5,7,28,33,34,35,36,37,38,39,40]. Lineages A and B were described as having the greatest diversity of mammalian hosts and vectors. Lineage A was reported in bats of Central Brazil and the Amazon [6,39] and in primates and marsupials from the Amazon region [5,29]. Lineage B was originally reported in the Amazon, infecting non-human primates, triatomines, sloths, anteater, and humans [27,28,29], and recently in the Atlantic Forest infecting a bat species [37]. *Rhodnius* *robustus* was found harboring lineage A in the Amazon biome [28]. Lineage C is not reported in Brazil but in countries such as Colombia, Panamá, Costa Rica, and El Salvador [27,28]. Lineage D was only reported in the Atlantic Forest, first in the rodent species *Echimys dasythrix* and then in a bat species (*Carollia perspicillata*) [36,37,39]. Lineage E was described only in bats from Central Brazil and *R. pictipes* from the Amazon region [6].

In this context, even though *T. rangeli* is one of the most studied trypanosomatids, previous studies demonstrated only a small piece of what occurs in nature. We started from the hypothesis that *T. rangeli* is a parasite with wide dispersion in nature and that is transmitted among animals that use all forest strata, and that is probably transmitted by other triatomines besides *Rhodnius*. Our study included samples from a much larger number of mammal species from a wide geographical range and identified new hosts, and extended the known geographical distribution of *T. rangeli* and its lineages. Since Brazil is a country of continental proportions, we were able to survey several types of landscapes. We characterized through the small subunit (SSU) rDNA barcoding 57 samples of *T. rangeli* deposited in our DNA collection (http://coltryp.fiocruz.br/) (accessed on 31 March 2021). These *T. rangeli* samples were obtained from hemocultures and blood clots of free-ranging wild animals and domestic dogs in five different biomes over a period of 12 years. This is the first time that such a large number of *T. rangeli* samples were evaluated in Brazilian biomes, and the data generated will improve the understanding of this parasite’s ecology.

## 2. Results

From a total of 1392 free-ranging wild mammals examined between 2005 and 2017, *Trypanosoma rangeli* was confirmed in 57 DNA samples (4.1%) (Table 1). We have demonstrated that *T. rangeli* is a widely dispersed multi-host trypanosomatid since it was found infecting 15 mammal species of six orders dispersed in five different Brazilian biomes (Table 1). *Trypanosoma rangeli* infection was observed for the first time in the raccoon species *Procyon cancrivorus* (n = 1), in a giant armadillo, *Priodontes maximus* (n = 1), in a howler monkey, *Alouatta belzebul* (n = 1), in capuchin monkeys, *Sapajus libidinosus* (n = 15), and in the rodent *Trinomys dimidiatus* (n = 1). Blood samples of the giant armadillo (C752, C776, and C792 samples) collected at different periods (June, October, and December 2017) were all positive for *T. rangeli* (Supplementary Materials Table S1). The Pantanal biome had the largest number of *T. rangeli* occurrences in mammals, followed by the Cerrado-Amazon transition area and Atlantic Forest (Table 1).

*Nasua nasua* (coatis) and capuchin monkeys had the highest number of individuals infected with *T. rangeli* (22/189 (11.6%) and 15/46 (32.6%), respectively) (Table 1). According to both t-tests, *T. rangeli* infection was statistically significant for the mammals analyzed (Tables S2 and S3).

**Figure 1:**
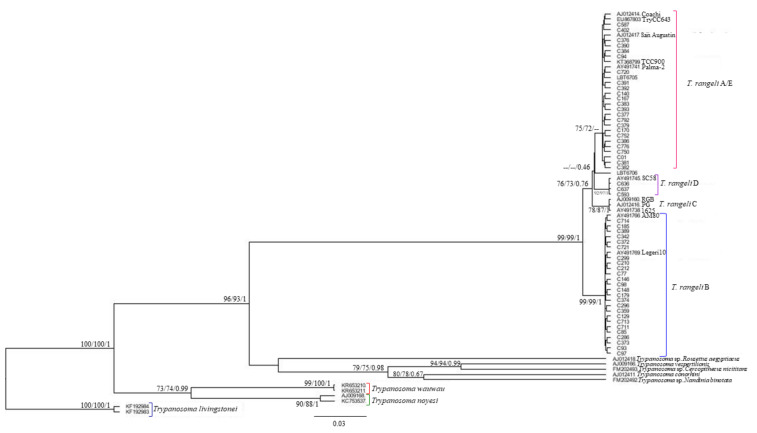
SSU rDNA reconstruction and phylogenetic trees based on 554 bp fragment alignment. Maximum likelihood ultrafast bootstrapping, SH-aLRT values, and Bayesian posterior probabilities are given near the nodes. Four *T. rangeli* lineage groups are shown: *T. rangeli* A/E; *T. rangeli* D; *T. rangeli* C; and *T. rangeli* B. The LBT6706 sequence is grouped separately from the *T. rangeli* A/E branch. The dashes represent lower or absent ultrafast bootstrap values, SH-aLRT values, and posterior probability values.

Fifty-four *T. rangeli* sequence samples were subjected to phylogenetic reconstruction by maximum likelihood (ML) and Bayesian inference (BI) (Figure 1). The sequences presented a base pair (bp) range from 556 to 912 nucleotides, and the final alignment presented 554 bp. Samples C375 (lineage A), C710, and RM2028 (lineage B) were not included in the phylogenetic reconstruction because they presented sequences with fewer than 554 nucleotides (441, 415, and 394 bp, respectively); thus, their lineage classification was performed solely based on BLAST screening. Both the ML and BI phylogenetic tree reconstructions grouped *T. rangeli* sequences into four lineage groups instead of five (Figure 1), as the A and E lineages were grouped together in the same clade, with some branch support, as observed in the ultra-bootstrap and SH-aLRT values (>70). The other three clusters were formed by *T. rangeli* lineages B, C, and D, all with high ultra-bootstrap, SH-aLRT (>70), and posterior probability values (PP = 1.0) (Figure 1). The lineage B cluster was the most distant from the other lineages (99 and 1.0 for the ultra-bootstrap, SH-aLRT, and posterior probability values, respectively). Although the lineage C cluster was separated from the other lineages with some branch support (>70), the separation between clusters A/E, LBT6706, and lineage D was not supported in either the ML or BI phylogenetic reconstructions (PP < 0.46).

**Figure 2:**
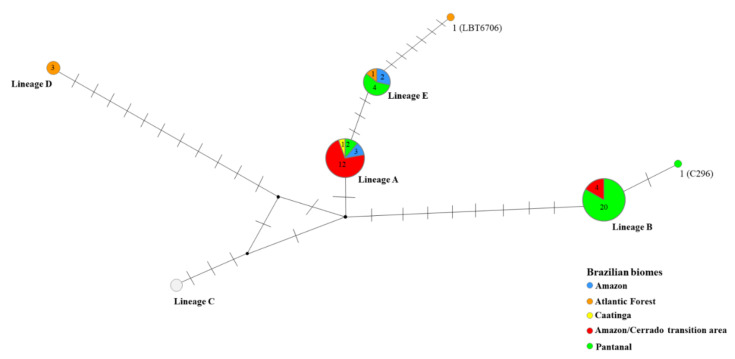
SSU rDNA sequence lineage network of *Trypanosoma rangeli* in different mammalian host species from Brazilian biomes. Networks were constructed with 65 SSU rDNA sequences, and the size of each node is proportional to the lineage frequency. The small black circle represents the median vector, which can be interpreted as an unsampled sequence or an extinct ancestral sequence.

The lineage network (Figure 2) was capable of separating *T. rangeli* into five groups corresponding to its lineages (A, B, C, D, and E). Two well-defined *T. rangeli* clusters were observed: one including lineage B and the other including the other lineages (Figure 2). The cluster related to *T*. *rangeli* lineage A was found circulating in three biomes and in the transition area between the Amazon and Cerrado biomes and was separated from lineage B with a single mutational step. Additionally, lineages A and E were demonstrated to be closely related since three polymorphic site mutations in 1087, 1113, and 1114 bp of SSU rDNA separated them. The lineage B node was separated from the other lineages by eight polymorphic sites, thus confirming it to be the most distant *T. rangeli* lineage from the others. The Pantanal biome presented the greatest occurrence of *T. rangeli* lineage B (Figure 2). Lineages B and C were also separated by four polymorphic sites. *Trypanosoma rangeli* lineage D was the most distant from lineages A and C, as it was separated by 11 mutation steps (Figure 2).

**Figure 3:**
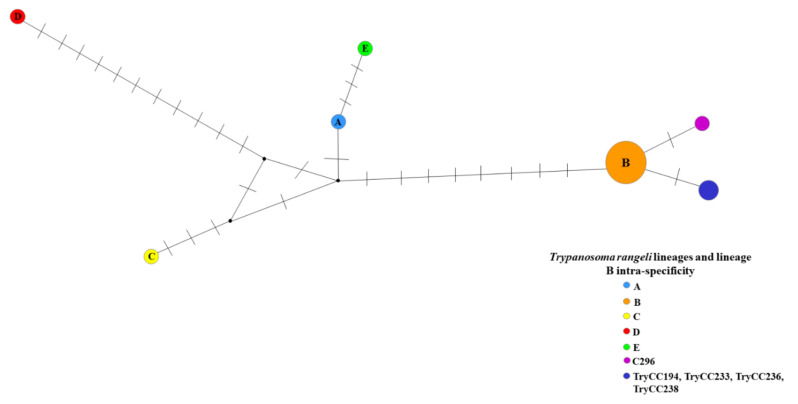
*Trypanosoma rangeli* lineage B intraspecific network. Networks were constructed with 25 SSU rDNA sequences. The small black circle represents the median vector, which can be interpreted as an unsampled sequence or an extinct ancestral sequence.

Two new sequences were observed separately from the other recognized lineages: one related to sample C296 (Amazon biome), which was clustered in lineage B in the phylogenetic analysis and separated from this lineage by a single polymorphic site (Figure 2 and Figure 3), and LBT 6706 (Atlantic Forest biome), which was separated from lineage E by six polymorphic sites (Figure 2). In *T. rangeli* lineage B, which is intraspecific (Figure 3), it is clear that the C296 sequence is different from the other *T*. *rangeli* B sequences that have already been described.

The predominant *T. rangeli* lineages were A and B (Table S1). Lineage A was shown to be the most widely distributed lineage, being detected in five Brazilian states in 7 different mammal species of five orders dispersed in the Caatinga, Pantanal, and Amazon biomes and in the Amazon-Cerrado transition area: Didelphimorphia (n = 2), Carnivora (n = 2), Chiroptera (n = 1), Primates (n = 1), and Rodentia (n = 1) (Figure 1).

**Figure 4:**
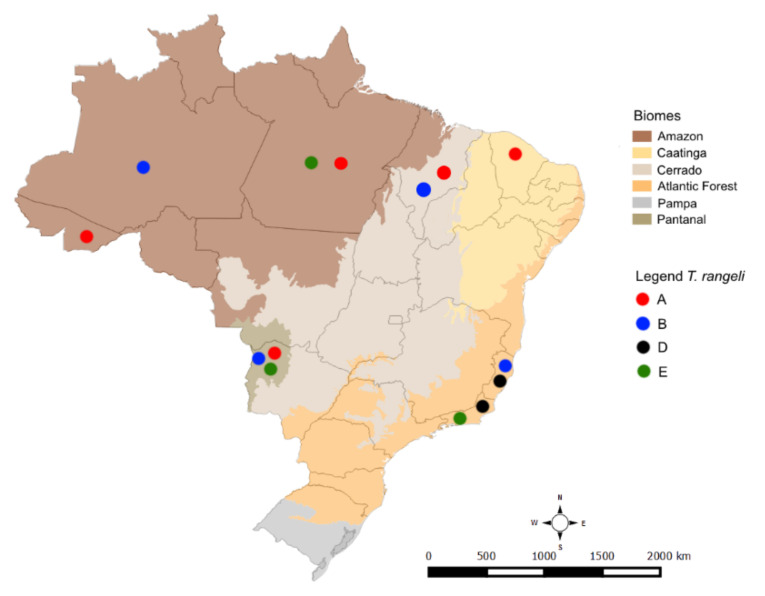
*Trypanosoma rangeli* lineage distribution map in mammalian hosts in different Brazilian biomes. The lineages are represented by dots of the following colors: red—lineage A, blue—lineage B, black—lineage D, and green—lineage E.

Figure 4 depicts the distribution of *T. rangeli* lineages in Brazilian biomes: lineages A and B were observed in four biomes, and lineage E was reported in three biomes (Amazon, Atlantic Forest, and Pantanal). Lineage A was the only *T. rangeli* lineage reported in the Caatinga biome (Figure 2 and Figure 4). The occurrence of lineage D was limited to the Atlantic Forest (Figure 2 and Figure 4). Four mammalian species presented infection by more than one lineage. The bat species (*Carollia perspicilata*) presented infections by the greatest diversity of *T. rangeli* lineages (A, B, and D). *Sapajus libidinosus* and *N. nausa* were infected by lineages A and B, and *C*. *familiaris* was infected by lineages A and E.

## 3. Discussion

In this study, we provide an overview of the *T. rangeli* enzootic scenario in Brazil. *T. rangeli* is a generalist trypanosome that is capable of infecting a broad range of mammalian species. *Trypanosoma rangeli* infection has been observed in six mammalian orders, Didelphimorphia, Chiroptera, Carnivora, Rodentia, Cingulata, and Primates, of five Brazilian biomes, thus confirming its generalist characteristics concerning host range. All these mammalian orders were demonstrated to be involved in parasite transmission in the different Brazilian biomes because animals with positive hemocultures suggest competence to be a source of *T. rangeli* infection of the vectors. Here, the discovery of *T. rangeli* infecting free-ranging mammals that use diverse habitats provides evidence that *T. rangeli* is circulating in different forest strata and that these animals are possibly interacting and dispersing the parasite within these areas. In addition, animals may acquire the infection either through the contaminating vectoral route or through the oral route through triatomine ingestion.

According to our results, the carnivore coati (*N. nasua*) and capuchin monkey (*S. libidinosus*) were the key hosts of *T. rangeli* in the Pantanal biome and in the transition area between the Cerrado and Amazon biomes, respectively. Therefore, in this situation, these mammals could be considered a transmission hub of the parasite. The key host transmission role played by these two mammalian taxa can be explained by their habits. Coatis are known to present diurnal, scansorial, and sociable habits, except for adult males, which are solitary [41,42,43,44]. This mammalian taxon uses the ground and tree canopies, where they build nests to rest and reproduce [45]. Coatis present omnivorous feeding behavior, predominantly ingesting invertebrates and fruit, but it is possible that it feeds by vertebrates and carrion [44,46].

The capuchin monkey displays arboreal habits, although the young monkeys go to the ground to play and live in small groups [47,48,49,50,51]. Moreover, they can also use the ground in the Pantanal biome [52]. Capuchin monkeys are considered a generalist opportunistic species and present a variable diet that includes primarily fruits; however, they also feed on seeds, nuts, flowers, gums, nectar, fungi, sap, eggs, insects, small vertebrates, and even some oysters and crab species in mangrove regions [48,53]. Here, we also increased the number of monkey species known to be infected with *T*. *rangeli*. This clarifies why *T. rangeli* is so often found in monkeys, as these species consume insects as a part of their diet, and we can speculate that these primates might be infected by *T. rangeli* through the oral route.

*Trypanosoma rangeli* was found in four different marsupial species in our study. Infection in marsupials was previously observed in the Amazon and Cerrado biomes in *D. albiventris* and *D. marsupialis* [15,54,55] and in Ecuador in *D. marsupialis* [56]. It is interesting to note that only a few individuals of *Didelphis* sp. were found with positive hemocultures for *T*. *rangeli*. These low positive hemoculture rates may be due to very low parasitemia and may underestimate the real rate of infection, as this non-sensitive parasitological method does not detect cryptic infections. Although we report other marsupial species (*Philander opossum* and *Didelphis aurita*) infected with *T. rangeli*, we expected a greater number of positive samples because marsupials are usually found to be infected with a diversity of trypanosomatid species, which probably would include *T. rangeli*. In relation to lineage occurrence, we observed marsupials infected with lineage A, as has already been observed [15,28], but here, we also observed marsupials infected with lineage D in the Atlantic Forest. Marsupials are described as bio-accumulators of *Trypanosoma* species because these animals are able to harbor high *Trypanosoma* species diversity [57,58]. The occupation of generalist habitats by marsupials may explain why these animals are infected with trypanosomatids, including *T. rangeli*.

*Trypanosoma rangeli* has already been shown to infect several rodent species: *Phyllomys dasythrix* in Brazil [39] and *Rhipidomys* spp., *Sciurus stramineus* [56], and *Oryzomys xanthaeolus* in Ecuador [59]. We added two more rodent species as hosts of *T. rangeli* in Brazil: *Coendou prehensilis* and *Trinomys dimidiatus*. Genera of the orders *Trinomys* and *Coendou* have terrestrial and arboreal habits, respectively [60]; *C*. *prehensilis* was considered exclusively herbivorous (consuming fruits and seeds) [61,62], but fruits and seeds may easily be contaminated by infected triatomine. The *Trinomys* diet can be frugivorous, granivorous, herbivorous, or insectivorous [43,63]. It is important to highlight that the *Trinomys* genus uses hollow trees and burrows in the ground for shelter while resting [63], and these areas can harbor *Trypanosoma*-infected triatomines.

Additionally, for the first time, we demonstrated *T. rangeli* infection in a giant armadillo (*Priodontes maximus*) from the Pantanal biome. This is the first report of a giant armadillo infected with lineage E. These animals dig and live in burrows that can be used by a variety of vertebrates and insects [40,64]. The authors also surmised that the micro-environment inside these burrows can provide favorable conditions for parasite and vector survival and proliferation. *P. maximus* ingests insects in its diet [65,66], which can also lead to *T. rangeli* infection, as has already been suggested in the case of *T*. *cruzi* [40,67]. One of these scenarios may have led to *T. rangeli* infection in the giant armadillo. This shows the different manners by which an animal can become infected and how complex the transmission cycle of *T. rangeli* can be. In addition, the same armadillo demonstrated a stable *T. rangeli* infection since, in the present study, three positive samples were obtained in different time periods. We do not consider this situation a reinfection since the DNA sequences of the three samples were identical, and other *T. rangeli* lineages circulate in this biome.

In the case of the giant armadillo infection, our data support the maintenance of the same parasite population and a state of high parasitemia over an extended period. Although there are studies showing that infection can be maintained for a long time [10], it is still unknown how *T. rangeli* multiplies in or invades vertebrate host cells [2,10,68,69,70]. Additionally, as an extracellular parasite, how it escapes the host’s antibody-based immune response are among questions that need answering to obtain a better understanding of *T. rangeli* ecology.

We have reported only three fruit-eating bats (*C. perpicillata*) infected with *T. rangeli*. Bats are known to be hosts of several trypanosomatid species and are probably the original hosts of the *T. cruzi* clade [36,37,38,71,72,73,74]. Other authors have also detected low *T. rangeli* infection rates in bats [6,38,56,75]. It is intriguing to find that *T. rangeli* infection is rare in bats. Bats usually feed on insects and small vertebrates, and they groom and regurgitate food [76,77,78], so they are frequently in contact with *Trypanosoma* spp.; due to these habits, they were supposed to have a high rate of exposure to *T. rangeli*. Experimentally, it was demonstrated that bats acquired *T*. *rangeli* infection after triatomine blood feeding [79], so transmission occurred through metacyclic forms of the parasite present during salivary inoculation. We can speculate about some explanations for this finding: (i) bats can rapidly control *T. rangeli* infection; (ii) they present some sort of unknown mechanism that prevents the infection from being established; or (iii) they present cryptic infections that are not detectable by hemocultural exams.

*Trypanosoma rangeli* infection in dogs has been reported in some Latin American countries [80,81]. In Brazil, dog infection by *T. rangeli* was observed for the first time in the Amazon [82], and we are reporting it for the first time in the Atlantic Forest. Lineages A and C were reported in dogs from Venezuela and Colombia, respectively [28]. Here, we added another lineage encountered in dogs; *T. rangeli* lineage E infection in dogs may be associated with the fact that these animals prey on insects or even small mammals that may be infected. Although these dogs were examined in the domicile environment, the dogs in rural areas on Amazon and Atlantic Forest live in houses that are located at the border and, in several cases, inside the forest, and they are circulating in the sylvatic environment most of the time. That is why we correlate the dogs with the sylvatic area.

Due to its wide distribution and broad range of mammalian host species, *T. rangeli* probably infects triatomine vectors other than *Rhodnius* spp., as has been suggested previously. This parasite transmission is correlated to *Rhodnius* species [7,83] because they present *T. rangeli* infective forms in salivary glands [8,33,84]. *Rhodnius* species occur in all the Brazilian biomes, with some species more common and well distributed in the Amazon biome, whereas other species occur in the Pantanal, Cerrado, Caatinga, and Atlantic Forest biomes [85,86]. Although the important role played by *Rhodnius* triatomine species in *T. rangeli* transmission is clear, other triatomine species have been reported: *Panstrongylus megistus* in Minas Gerais and Santa Catarina states [15,21] and *Triatoma brasiliensis* in the Brazilian semi-arid region [87]. Although DNA from *T. rangeli* has been reported in the digestive tract of *T. brasiliensis*, the possibility of an animal ingesting and becoming infected from parasitic forms of triatomine cannot be ruled out. Despite the occurrence of *R. domesticus* in the Atlantic Forest [85], it is not common to find this species in the sylvatic environment. Dario and coworkers [37] suggested that *Triatoma* species might be responsible for *T. rangeli* transmission in Espírito Santo state (Atlantic Forest). In the Atlantic Forest of Santa Catarina, *Panstrongylus megistus* has already been found to be infected by *T. rangeli* [21], showing that other species can be involved in transmission. In Rio de Janeiro, the situation might be similar to that observed in Espírito Santo state, where *R. domesticus* is reported [85]. Therefore, it is likely that triatomine species other than *Rhodnius* sp. are also able to transmit this trypanosomatid. In areas where *Rhodnius* presence is confirmed, we suggest that infection might be occurring by the inoculative vectorial route, with contaminative *T. rangeli* forms present in the salivary gland. In the Atlantic Forest, where *Rhodnius* species are not found frequently and another genus of triatomine has been found to cause infection [15,21], another *T. rangeli* transmission route may occur.

Here, we present *T. rangeli* occurrence in mammals from a new biome—the Caatinga—increasing its distribution beyond the Amazon, Atlantic Forest, Cerrado, and Pantanal biomes. Therefore, *T. rangeli* is distributed in almost all Brazilian biomes. The Pantanal biome, in this study, was the one with the highest total number of infected animals: *N. nasua*, *P. cancrivorus*, and *P. maximus*. This contradicts the statement of other authors that most cases of *T. rangeli* infection occur in the Amazon biome [32], where four mammal species were found infected: *D. marsupialis*, *S. b. bicolor*, *C. familiaris,* and *C. perspicillata*. Although the Pantanal presented more mammals infected with *T. rangeli*, most of them were observed in coatis; in the Amazon, four different species were found infected, showing the broad range of infected species in this biome. Lineage A was found most often, lineage B was found only in the Amazon region [27,28], and lineage E was observed for the first time in the Pantanal biome [6]. We have demonstrated broad-spectrum circulation since these lineages were observed in different biomes than those in which they were first described. Lineage D was not observed outside the Atlantic Forest, but its occurrence was observed in distant forest fragments, leading to the conclusion that this *T. rangeli* lineage has an extensive distribution and probably can be observed in other biomes. In light of these findings, previous suggestions that *T. rangeli* lineages are linked to certain biomes or areas are not supported, but further research is required.

Intraspecific studies have demonstrated the diversity of the *Trypanosoma* genus, and the species of this genus were eventually subdivided into genotypes, as already noted, that include *T*. *cruzi* [88], *T. rangeli* [5,6,7,23,27,28], and some *Trypanosoma* species from bats [57,73]. In the case of *T. rangeli*, intra-specificity was observed across a variety of molecular markers, such as spliced leaders [89], random amplified polymorphic DNA (RAPD) [5,21], kDNA [23,24,25], and histone H2A genes [90], from which it was possible to differentiate this species into two or three groups. Maia da Silva et al. [5], using the RAPD assay for 22 *T. rangeli* samples, were able to separate this species into four lineages: A, B, C, and D. This same division was observed when 34 *T. rangeli* samples were subjected to molecular characterization using two different regions of the 18S rDNA: SSU and ITS1 [27]. These four lineages were corroborated by spliced leader RNA gene sequences [28], which confirmed these sequences as the most valuable and sufficiently defined reliable *T. rangeli* lineages [25,28,89]. Lineage E was described later [6] using spliced leader sequences and corroborated using other sequences [30,31,32]. In addition, Espinosa-Álvarez et al. [32] used 800 bp sequences from the V7-V8 region of SSU rDNA concatenated with gGAPDH and were able to cluster *T. rangeli* into the same five lineages. This subdivision is also possible using cathepsin L-like proteases and proline-racemase pseudogenes [30,31]. In our study, we were able to separate *T*. *rangeli* into four groups (A/E, B, C, and D) using a smaller SSU rDNA fragment (554 bp), but we were not able to separate lineages A from E in the phylogenetic tree reconstruction using only SSU rDNA. However, using the haplotype network, it was possible to separate all the lineages.

We observed two different sequences of *T. rangeli*, demonstrating the occurrence of new genotypes that may correspond to new lineages. The two samples showing different SSU rDNA sequences were from hosts collected in the Atlantic Forest and Pantanal biomes, which have high biodiversity [91,92]. We can affirm that this biodiversity extends to parasites, not only for the *T. rangeli* lineages but also for other trypanosomatid species and lineages, as previously reported [36,37,93]. These results reinforce that the Atlantic Forest is a biodiversity hotspot at all levels, from macro- to microorganisms, and that we are probably dealing with a new parasite hotspot.

We are not proposing any new genotype classification because SSU rDNA sequences alone should not be employed for reliable descriptions of new lineages. The subdivision of *T. rangeli* into two main groups as initially proposed [94] or into five or more lineages must be supported by multiple genetic or phylogenetic analyses. All the groups contain members with different host species (human, domestic or sylvatic mammals) without any vertebrate host species associations. Grouping was independent of mammalian host species and geographical origin, indicating that other factors determine this segregation. Some associations (not strict) with the *Rhodnius* species complex have been suggested. However, both the taxonomy and evolutionary history of Rhodnini are far from being clearly understood, and any vector-parasite associations must be interpreted with caution [25,28,32,94]. Most of the studies, including ours, involve subsampling, and a final division/classification of *T*. *rangeli* genotypes needs more representative sampling.

To conclude, this study shows how much is still unknown about *T. rangeli*. Knowledge of mammalian hosts and vector ranges and *T*. *rangeli* infection stability in these mammalian hosts and lineage classification is still far from complete and will likely expand in the future as wider geographic areas are explored.

## 4. Material and Methods

### 4.1. Trypanosoma Rangeli Study Areas

**Figure 5:**
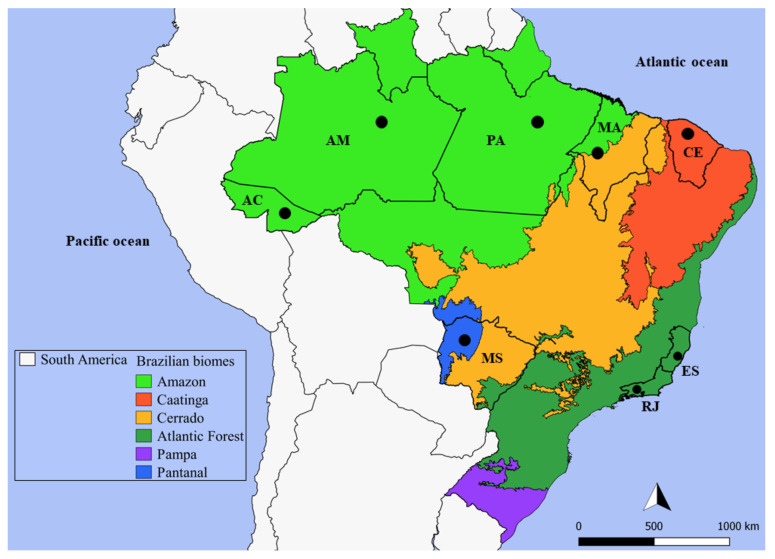
Brazilian biomes investigated for *Trypanosoma* spp. infection in mammals. Six biomes are represented: Amazon, Cerrado, Caatinga, Atlantic Forest, Pantanal, and Pampa. The acronyms represent the Brazilian states, and the black dots represent the places where fieldwork was performed: AC—Acre, AM—Amazonas, CE—Ceará, ES—Espírito Santo, MA—Maranhão, MS—Mato Grosso do Sul, PA—Pará, and RJ—Rio de Janeiro.

The fieldwork, the aim of which was to study mammalian trypanosomatids, was conducted by our group from 2005 to 2017 [37,38,57,95,96,97] (Dario MA (data not shown) and Lisboa CV (data not shown)). The data are summarized in Figure 5 and Table 2.

### 4.2. Trypanosoma Rangeli DNA Samples Origin

DNA samples of the trypanosomatids isolated from hemocultures (n = 55) and blood clots of two animals were deposited in the DNA library of the *Trypanosoma* from wild and domestic mammals and vectors collection (COLTYP/Fiocruz). The hemocultures were washed with phosphate-buffered saline solution, 100 µg/mL proteinase K (Invitrogen, Carlsbad, CA, USA), and 0.5% sodium dodecyl sulfate was added, and the samples were incubated at 56 °C for 2 h [44]. After this step, the DNA was extracted by the phenol-chloroform method [98]. For the two blood clots, the absolute ethanol was removed, and 50 µL of each blood clot was transferred to a new 1.5 mL tube. DNA extraction was performed according to [58]. The DNA concentration and purity of the DNA samples were quantified using a NanoDrop 1000 Spectrophotometer (Thermo Scientific, Waltham, MA, USA).

### 4.3. Trypanosoma Rangeli Molecular Characterization

The DNA samples were subjected to two PCR methodologies for *T*. *rangeli* identification: (i) 15 samples underwent conventional PCR for amplification of the V7-V8 region of SSU rDNA [99], and (ii) 42 samples underwent nested PCR to amplify the SSU rDNA gene [100,101]. For amplification of the V7-V8 region, 8.5 µL of GoTaq MasterMix (Promega, Madison, WI, USA), 100 ng of DNA template, 10 pmol of the 609F (5′CACCCGCGGTAATTCCAGC3′), and 706R (5′CTGAGACTGTAACCTCAA3′) primers and sterile deionized water were used in a 25 µL final mix solution. Amplification was performed under the following conditions: an initial denaturation cycle at 94 °C for 3 min; 30 cycles at 94 °C for 1 min, 48 °C for 2 min and 72 °C for 2 min; and final elongation at 72 °C for 10 min.

For the nested PCR, 16 pmol of each external primer (TRY927F (5′GAAACAAGAAACACGGGAG3′) and TRY927R (5′CTACTGGGCAGCTTGGA3′)), 8.5 µL of GoTaq MasterMix (Promega, Madison, WI, USA), 50 ng of DNA template, and sterile deionized water (added to reach a final volume of 25 µL) were used. Amplification was performed under the following conditions: initial denaturation at 94 °C for 3 min; 30 cycles at 94 °C for 30 s, 55 °C for 60 s, and 72 °C for 90 s; and final elongation at 72 °C for 10 min. The first-round PCR products were diluted (1:10) in sterile deionized water. Two microlitres of this dilution was used as a template for the second round of PCRs using the internal primers SSU561F (5′TGGGATAACAAAGGAGCA3′) and SSU561R (5′CTGAGACTGTAACCTCAAAGC3′) under the same conditions applied in the first round. For the DNA samples from the blood clots, 12.5 5 µL of GoTaq MasterMix and 20 pmol of each primer were used. All the SSU rDNA amplifications were performed with a Swift MaxPro Thermal Cycle (Esco Scientific, Singapore).

Electrophoresis of the PCR products (~850 bp for the V7-V8 region and ~600 bp for the nested PCR) was performed in 2% agarose gels (Figure S1), which were stained with ethidium bromide solution and visualized under ultraviolet light. The amplified products were purified using the Illustra GFX PCR DNA and Gel Band Purification Kit (GE Healthcare Life Sciences, Little Chalfont, Buckinghamshire, UK). The SSU rDNA amplicons underwent Sanger DNA sequencing reactions for both DNA strands (V7-V8: 609F—5′CACCCGCGGTAATTCCAGC3′ and 706R—5′CTGAGACTGTAACCTCAA3′; SSU rDNA: SSU561F—5′TGGGATAACAAAGGAGCA3′ and SSU561R—5′CTGAGACTGTAACCTCAAAGC3′) with a BigDye Terminator v3.1 Cycle Sequencing Kit (Applied Biosystems, Foster City, CA, USA) on an ABI 3730 DNA sequencer available at the PDTIS/Fiocruz sequencing facilities.

### 4.4. Phylogenetic Analysis of Trypanosoma rangeli Lineages

To obtain the consensus SSU rDNA sequences, each forward and reverse sequence was assembled and edited using SeqMan (DNASTAR Lasergene, Gatc, Konstanz, Germany). *Trypanosoma rangeli* sequences obtained in this study were aligned to an SSU rDNA homologous region in *T. rangeli* lineages and outgroup sequences deposited in GenBank (Table S2) using the L-INS-i algorithm in MAFFT v.7.0 [102]. The obtained alignment was visualized and manually edited on MegaX software [103]. To determine the *T. rangeli* lineages, maximum likelihood (ML) estimation and Bayesian inference (BI) were performed. The equal-frequency transition model with gamma-distributed rate variation among sites (TIM2ef+G) was the best model for the dataset, as indicated by the corrected Akaike information criterion (AICc) and Bayesian information criteria (BIC) obtained in jModelTest v.2 [104]. The ML tree reconstruction was performed in the IQ-Tree program [105,106] available on PhyloSuite v.1.2.2. For branch support, ultrafast bootstrapping [107] was performed with 5000 replicates with 1000 maximum interactions and 0.99 minimum correlation coefficients. To validate the ultrafast bootstrap results, the SH-aLRT branch test with 5000 replicates was also applied.

Bayesian tree reconstruction was performed in Bayesian Evolutionary Analysis Sampling Trees (BEAST) v2.6.2 [108]. The Bayesian Markov chain Monte Carlo (MCMC) method was used to assign *T. rangeli* lineages prior to information. The birth-death model specification was used in tree reconstruction. Four independent runs were performed for 20 M with sampling every 2000 generations. The runs converged, and the effective sample size (ESS) was calculated after 10% of each run was excluded (burn-in) from each run in TRACER v.1.6 [109]. The parameters selected led to ESSs higher than 500 and were considered appropriate. The final tree was generated with maximum clade credibility (MCC) based on 32,404 trees (burn-in = 3600) and a 0.6 posterior probability limit (PP) in Tree Annotator. Both the ML and BI reconstructions were visualized in Figtree v.1.4.3.

Two haplotype networks based on only *T. rangeli* samples were generated in Network software version 5.0.1.1 (fluxus-engineering.com) to define evolutionary relationships among the *T. rangeli* lineages and to observe the intra-specificity of lineage B. The SSU rDNA lineage and lineage B intra-specificity networks were built using median-joining [110] and maximum parsimony [111] post-processed clean-up procedure. The SSU rDNA sequences used in the phylogenetic reconstruction and in the haplotype networks are listed in Table S4.

### 4.5. Statistical Analysis

The overall infection rate and the infection rate for each mammalian species were calculated using the number of species positive for *T. rangeli* infection divided by the total number of analyzed mammals. In addition, a two-tailed t-test was performed to verify the significance level of infection in mammals. T-tests of paired sample averages and two samples assuming different variances were performed with a significance level of *p* < 0.05. All the statistical analyses were performed in Microsoft Excel^®^ 365.

## Figures and Tables

**Table 1:** *Trypanosoma rangeli* infection was demonstrated in different mammalian species in Brazilian biomes between 2005 and 2017.

Order	Species	*T. rangeli* Infection/Total Examined	Infection Rate (%)	Biome	*T. rangeli* Lineage
Carnivora	*Nasua nasua*	22/189	11.6	Pantanal	A and B
	*Canis familiaris*	4/119	3.4	Amazon Forest (n = 2)	A and E
				Atlantic Forest (n = 2 *)	E
	*Procyon cancrivorus*	1/17	5.9	Pantanal	E
Cingulata	*Priodontes maximus*	3 **/10	30	Pantanal	E
Chiroptera	*Carollia perspicillata*	3/279	1.1	Amazon Forest (n = 1)	A
				Atlantic Forest (n = 2)	B and D
Didelphimorphia	*Didelphis albiventris*	1/303	0.3	Caatinga	A
	*Didelphis aurita*	1/271	0.4	Atlantic Forest	D
	*Didelphis marsupialis*	1/58	1.7	Amazon Forest	A
	*Philander opossum*	1/59	1.7	Amazon Forest	E
Primates	*Alouatta belzebul*	1/6	16.7	Amazon-Cerrado transition area	B
	*Alouatta caraya*	1/5	20	Amazon-Cerrado transition area	B
	*Sapajus libidinosus*	15/46	32.6	Amazon-Cerrado transition area	A and B
	*Saguinus bicolor bicolor*	1/24	4.2	Amazon	B
Rodentia	*Coendou prehensilis*	1/5	20	Amazon-Cerrado transition area	A
	*Trinomys dimidiatus*	1/1	100	Atlantic Forest	D
	Total	57/1392	4.1%	-	-

* The two samples from the Atlantic Forest were characterized from blood clot samples. ** The three *T. rangeli* infections from *P. maximus* are from the same animal collected at different time points.

**Table 2:** Mammal species captured during fieldwork performed in different Brazilian biomes while surveying for *Trypanosoma rangeli* between 2005 and 2017.

Order	Species	Number of Samples
Carnivora	*Nasua nasua*	189
	*Canis familiaris*	119
	*Procyon cancrivorus*	17
Cingulata	*Priodontes maximus*	10
Chiroptera	*Carollia perspicillata*	279
Didelphimorphia	*Didelphis albiventris*	303
	*Didelphis aurita*	271
	*Didelphis marsupialis*	58
	*Philander opossum*	59
Primates	*Alouatta belzebul*	6
	*Alouatta caraya*	5
	*Sapajus libidinosus*	46
	*Saguinus bicolor bicolor*	24
Rodentia	*Coendou prehensilis*	5
	*Trinomys dimidiatus*	1
Total	15	1392

## Data Availability

The data presented in this study are openly available in the GenBank database (https://www.ncbi.nlm.nih.gov/genbank/) (accessed on 31 March 2021). The SSU rDNA accession numbers are provided in Table S1.

## Supplementary Materials

The following are available online at https://www.mdpi.com/article/10.3390/pathogens10060736/s1. Table S1: *Trypanosoma rangeli* infection in different mammalian species in Brazilian biomes. Table S2: Two-tailed t-tests of paired average samples of *Trypanosoma rangeli* infection in mammals. Table S3: Two-tailed t-test of two samples of *Trypanosoma rangeli* infection assuming different variances. Table S4: Trypanosomatid SSU rDNA sequences retrieved from GenBank used for phylogenetic analysis. Figure S1: Representative 2% agarose gel electrophoresis of 18S rDNA molecular markers for Trypanosoma rangeli molecular identification.
