# Supplementary material for: Trypanosoma rangeli Genetic, Mammalian Hosts, and Geographical Diversity from Five Brazilian Biomes
Source: Pathogens. 2021 Jun 11;10(6):736. doi: 10.3390/pathogens10060736 (PMC8230690; doi:10.3390/pathogens10060736)

Table S1: *Trypanosoma rangeli* infection in different mammalian species in Brazilian biomes.

Sample ID	Host	Year	Biome	SSU rDNA	GenBank accession number
C01	*Didelphis albiventris*	2005	CE/Caatinga	*T. rangeli* A	MN648976
C140	*Nasua nasua*	2009	MS/Pantanal	*T. rangeli* A	MN648980
C167	*Didelphis marsupialis*	2009	PA/Amazon	*T. rangeli* A	MN649025
C375	*Sapajus libidinosus*	2011	MA/Amazon-Cerrado transition area	*T. rangeli* A	MN648994
C376	*Sapajus libidinosus*	2011	MA/Amazon-Cerrado transition area	*T. rangeli* A	MN648995
C377	*Sapajus libidinosus*	2011	MA/Amazon-Cerrado transition area	*T. rangeli* A	MN648996
C379	*Sapajus libidinosus*	2011	MA/Amazon-Cerrado transition area	*T. rangeli* A	MN648997
C381	*Sapajus libidinosus*	2011	MA/Amazon-Cerrado transition area	*T. rangeli* A	MN648998
C382	*Sapajus libidinosus*	2011	MA/Amazon-Cerrado transition area	*T. rangeli* A	MN648999
C383	*Sapajus libidinosus*	2011	MA/Amazon-Cerrado transition area	*T. rangeli* A	MN649000
C384	*Sapajus libidinosus*	2011	MA/Amazon-Cerrado transition area	*T. rangeli* A	MN649001
C386	*Coendou prehensilis*	2011	MA/Amazon-Cerrado transition area	*T. rangeli* A	MN649002
C390	*Sapajus libidinosus*	2011	MA/Amazon-Cerrado transition area	*T. rangeli* A	MN649004
C391	*Sapajus libidinosus*	2011	MA/Amazon-Cerrado transition area	*T. rangeli* A	MN649005
C392	*Sapajus libidinosus*	2011	MA/Amazon-Cerrado transition area	*T. rangeli* A	MN649006
C393	*Sapajus libidinosus*	2011	MA/Amazon-Cerrado transition area	*T. rangeli* A	MN649007
C402	*Sapajus libidinosus*	2011	MA/Amazon-Cerrado transition area	*T. rangeli* A	MN649008
C587	*Carollia perspicillata*	2014	AC/Amazon	*T. rangeli* A	KY649114[a]
C750	*Canis familiaris*	2017	AC/Amazon	*T. rangeli* A	MN649017
C77	*Nasua nasua*	2009	MS/Pantanal	*T. rangeli* B	MN648977
C85	*Nasua nasua*	2009	MS/Pantanal	*T. rangeli* B	MN648978
C93	*Nasua nasua*	2009	MS/Pantanal	*T. rangeli* B	MN649021
C97	*Nasua nasua*	2009	MS/Pantanal	*T. rangeli* B	MN649023
C98	*Nasua nasua*	2009	MS/Pantanal	*T. rangeli* B	MN649024
C129	*Nasua nasua*	2009	MS/Pantanal	*T. rangeli* B	MN648979
C146	*Nasua nasua*	2009	MS/Pantanal	*T. rangeli* B	MN649027
C148	*Nasua nasua*	2009	MS/Pantanal	*T. rangeli* B	MN648981
C179	*Nasua nasua*	2007	MS/Pantanal	*T. rangeli* B	MN648982
C185	*Nasua nasua*	2009	MS/Pantanal	*T. rangeli* B	MN648983
C210	*Nasua nasua*	2010	MS/Pantanal	*T. rangeli* B	MN648984

C212	*Nasua nasua*	2010	MS/Pantanal	*T. rangeli* B	MN648985
C286	*Nasua nasua*	2006	MS/Pantanal	*T. rangeli* B	MN648986
C296	*Nasua nasua*	2006	MS/Pantanal	*T. rangeli* B	MN648987
C299	*Nasua nasua*	2008	MS/Pantanal	*T. rangeli* B	MN648988
C342	*Nasua nasua*	2008	MS/Pantanal	*T. rangeli* B	MN648989
C359	*Nasua nasua*	2011	MS/Pantanal	*T. rangeli* B	MN648990
C372	*Alouatta belzebul*	2011	MA/Amazon-Cerrado transition area	*T. rangeli* B	MN648991
C373	*Alouatta caraya*	2011	MA/Amazon-Cerrado transition area	*T. rangeli* B	MN648992
C374	*Sapajus libidinosus*	2011	MA/Amazon-Cerrado transition area	*T. rangeli* B	MN648993
C389	*Sapajus libidinosus*	2011	MA/Amazon-Cerrado transition area	*T. rangeli* B	MN649003
C710	*Saguinus bicolor bicolor*	2016	AM/Amazon	*T. rangeli* B	MN649011
C711	*Nasua nasua*	2016	MS/Pantanal	*T. rangeli* B	MN649012
C713	*Nasua nasua*	2016	MS/Pantanal	*T. rangeli* B	MN649013
C714	*Nasua nasua*	2016	MS/Pantanal	*T. rangeli* B	MN649014
C721	*Nasua nasua*	2016	MS/Pantanal	*T. rangeli* B	MN649016
RM2028	*Carollia perspicillata*	2015	ES/Atlantic Forest	*T. rangeli* B	MF141861[a]
C593	*Carollia perspicillata*	2014	ES/Atlantic Forest	*T. rangeli* D	MF141848[a]
C636	*Trinomys dimidiatus*	2015	RJ/Atlantic Forest	*T. rangeli* D	MN649009
C637	*Didelphis aurita*	2015	RJ/Atlantic Forest	*T. rangeli* D	MN649010
C94	*Canis familiaris*	2009	PA/Amazon	*T. rangeli* E	MN649022
C170	*Philander opossum*	2008	PA/Amazon	*T. rangeli* E	MN649026
C720	*Procyon cancrivorus*	2016	MS/Pantanal	*T. rangeli* E	MN649015
C752[b]	*Priodontes maximus*	2017	MS/Pantanal	*T. rangeli* E	MN649018
C776[b]	*Priodontes maximus*	2017	MS/Pantanal	*T. rangeli* E	MN649019
C792[b]	*Priodontes maximus*	2017	MS/Pantanal	*T. rangeli* E	MN649020
LBT 6705	*Canis familiaris*	2015	RJ/Atlantic Forest	*T. rangeli* E	MN661344
LBT 6706	*Canis familiaris*	2015	RJ/Atlantic Forest	*T. rangeli* E	MN661345

[a] sequence from previous studies.

[b] samples from the same specimen collected in different periods of time.

Brazilian states: AC - Acre, AM - Amazonas, CE - Ceará, ES - Espírito Santo, MA Maranhão, MS - Mato Grosso do Sul, PA - Pará, RJ - Rio de Janeiro.

Table S2: Two-tailed t-tests of paired average samples of *Trypanosoma rangeli* infection in mammals.

	Infected	Total
Mean	3,8	92,8
Variance	38,31428571	12414,88571
Observations	15	15
Pearson's Correlation	0,17092652	
Mean difference hypothesis	0	
Gl	14	
Stat t	-3,118499881	
P(T<=t) two-tail	0,007549767	
critical two-tail t	2,144786688	

Table S3: Two-tailed t-test of two samples of *Trypanosoma rangeli* infection assuming different variances.

	Infected	Total
Mean	3,8	92,8
Variance	38,31428571	12414,88571
Observations	15	15
Mean difference hypothesis	0	
Gl	14	
Stat t	-3,088838165	
P(T<=t) two-tail	0,008007961	
critical two-tail t	2,144786688	

Table S4: Trypanosomatid SSU rDNA sequences retrieved from GenBank used for phylogenetic analysis

Sample	Location	Host	GenBank accession number
***Trypanosoma rangeli* A**			
**San Augustin***	Colombia	*Homo sapiens*	AJ012417
**Coachi**	Colombia	*Rhodnius prolixus*	AJ012414
**Palma-2**	Venezuela	*Rhodnius prolixus*	AY491741
***Trypanosoma rangeli* B**			
**Legeri10***	Brazil	*Tamandua tetradactyla*	AY491769
**AM80***	Brazil	*Homo sapiens*	AY491766
**Preguici***	Brazil	*Choloepus didactylus*	AY491767
**AM11***	Brazil	*Homo sapiens*	AY491758
**Legeri32***	Brazil	*Tamandua tetradactyla*	AY491759
**4176***	Brazil	*Rhodnius brethesi*	EF071580
**TryCC207***	Brazil	*Cebuella pygmaea*	AY491752
**TryCC194***	Brazil	*Cebuella pygmaea*	AY491753
**TryCC233***	Brazil	*Saguinus l. labiatus*	AY491756
**TryCC238***	Brazil	*Saguinus l. labiatus*	AY491754
**TryCC236***	Brazil	*Saguinus f. weddelli*	AY491755
**TryCC205***	Brazil	*Aotus sp*	AY491757
**TryCC416***	Brazil	*Alouatta stramineus*	AY491760
**TryCC427***	Brazil	*Callicebus lugens*	AY491751
***Trypanosoma rangeli* C**			
**PG***	Panama	*Homo sapiens*	AJ012416
**RGB**	Venezuela	*Canis familiaris*	AJ009160
**1625**	El Salvador	*Homo sapiens*	AY491738

Trypanosoma rangeli **D**			
**SC58***	Brazil	*Echimys dasythrix*	AY491745
Trypanosoma rangeli **E**			
**TryCC643***	Brazil	*Platyrrhinus lineatu*	FJ900242
**TCC900**	Brazil	*Rhodnius pictipes*	KT368799
**Outgroup species**			
Trypanosoma conorhini **USP**	Brazil	*Rattus rattus*	AJ012411
Trypanosoma **sp. NanDoum1**	Cameroon	*Nandinia binotata*	FM202492
Trypanosoma **sp. HochNdi1**	Cameroon	*Cercopithecus nictitans*	FM202493
T. vespertilionis **P14**	England	*Pipistrellus pipistrellus*	AJ009166
Trypanosoma **sp.**	Gabon	*Rousettus aegyptiacus*	AJ012418
Trypanosoma wauwau **CBT68**	Brazil	*Pteronotus parnellii*	KR653210
Trypanosoma wauwau **BMC 1069**	Brazil	*Pteronotus parnellii*	KR653211
Trypanosoma **sp. G8**	Australia	*Bettongia sp.*	KC753537
Trypanosoma noyesi **H25**	Australia	*Macropus giganteus*	AJ009168
T. livingstonei **1304**	Mozambique	*Rhinolophus landeri*	KF192983
T. livingstonei **1953**	Mozambique	*Hipposideros caffer*	KF192984

*Sequences used in the haplotype network analysis of *T. rangeli* lineage B intra-specificity.

Figure S1: Representative 2% agarose gel electrophoresis of 18S rDNA molecular markers for *Trypanosoma rangeli* molecular identification. The 2% agarose gel was stained with ethidium bromide, and 100 base-pair ladders were used: A) PCR product (~850 bp) for the V7-V8 region; B) PCR products for the SSU rDNA (~600 bp).

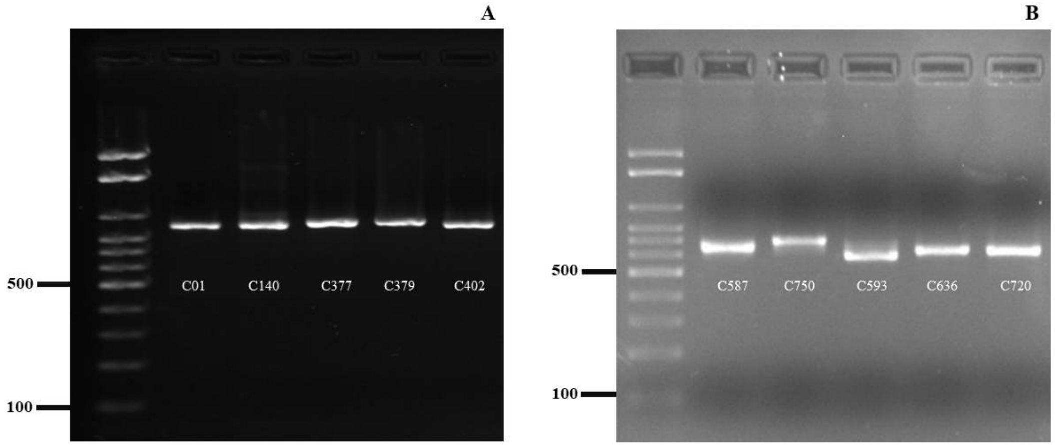

Supplement: Supplementary file 1 [file pathogens-10-00736-s001.zip › pathogens-1128035-supplementary.pdf]
